# Asthma and/or hay fever as predictors of fertility/impaired fecundity in U.S. women: National Survey of Family Growth

**DOI:** 10.1038/s41598-019-55259-8

**Published:** 2019-12-10

**Authors:** Paul C. Turkeltaub, Richard F. Lockey, Katie Holmes, Erika Friedmann

**Affiliations:** 1 Independent Scientist, PO Box 61571, Potomac, Maryland 20859 USA; 2 Division of Allergy & Immunology, University of South Florida College of Medicine, 13000 Bruce B. Downs Blvd, Tampa, Florida 33613 USA; 3 Organizational Systems and Adult Health, University of Maryland School of Nursing, 655 W. Lombard St., Baltimore, Maryland 21201 USA; 4 Present Address: The Hilltop Institute, University of Maryland Baltimore County, 1000 Hilltop Circle, Baltimore, Maryland 21250 USA

**Keywords:** Infertility, Asthma

## Abstract

This study addresses whether asthma and/or hay fever predict fertility and impaired fecundity. The lifetime number of pregnancies (fertility) and spontaneous pregnancy losses (impaired fecundity) in 10,847 women representative of the U.S. population 15 to 44 years of age with histories of diagnosed asthma and/or hay fever are analyzed in the 1995 National Survey of Family Growth using multivariable Poisson regression with multiple covariates and adjustments for complex sampling. Smokers have significantly increased fertility compared to nonsmokers. Smokers with asthma only have significantly increased fertility compared to other smokers. Higher fertility is associated with impaired fecundity (ectopic pregnancy, miscarriage, stillbirth). Women with asthma (with and without hay fever) have significantly higher pregnancy losses than women without asthma. With increasing number of pregnancies, smokers have increased pregnancy losses compared to nonsmokers. Smokers, especially those with asthma only, have increased fertility and require special attention as to their family planning needs, reproductive health, and smoking cessation. Women with asthma, regardless of number of pregnancies, and smokers with higher numbers of pregnancies have high risk pregnancies that require optimal asthma/medical management prenatally and throughout pregnancy. Whether a proinflammatory asthma endotype underlies both the increased fertility and impaired fecundity associated with age and smoking is discussed.

## Introduction

The increased prevalence of allergic respiratory disease worldwide^1–3^ is attributed in part to atopic women having more pregnancies resulting in live births than nonatopic women^4^ as both successful pregnancy^5,6^ and allergic diseas^7,8^ are associated with a type 2 (T2) immune response endotype i.e., group 2 innate lymphoid/adaptive T helper 2 cell immune response^9,10^. The National Survey of Family Growth (NSFG) is a cross-sectional survey representative of the U.S. population of women of reproductive age^11^. Questions pertaining to asthma and hay fever were requested by one of the authors (PCT) to be included in the 1995 NSFG to explore the relationship of allergic phenotype to fertility/fecundity. Fertility is defined as the capacity to establish a clinical pregnancy^12^. Fecundity is defined as the capacity to have a live birth^12^.

Although prior studies addressed fertility in women with asthma, these studies conceptualized fertility from different perspectives, analyzing live birth rates^13^, currently defined as fecundity^12^ or analyzing time to pregnancy >1 year^14^, currently defined as infertility^12^. While both of these approaches provide important information, they do not address the process leading from pregnancy to pregnancy loss and thus are not comparable to the current analysis.

We previously studied predictors of spontaneous pregnancy losses in women with asthma and/or hay fever based on their most recent singleton pregnancy^15^. The current study conceptualizes birth outcomes as a two-step process, becoming pregnant and carrying the pregnancy to term. Our previous study included only one pregnancy and its outcomes, it did not consider the possible contribution of asthma to the process of becoming pregnant or evaluate the contribution of the number of previous pregnancies to these outcomes. As a prior study indicated that the numbers of previous pregnancies increase the risk of pregnancy losses^16^, the current study analyzes the contributions of asthma and hay fever to the predictors of pregnancy loss controlling for the number of previous pregnancies.

## Materials and Methods

### NSFG design

The survey methodology is previously described^11^. In brief, data are drawn from the 1995 NSFG, a multistage probability sampling of the civilian non-institutionalized population of women 15 to 44 years of age, yielding estimates that are representative of the U.S population of women in this age range. The 1995 NSFG sample was drawn from a larger sample of households previously interviewed for the 1993 National Health Interview Survey (NHIS). A complex stratified cluster sampling design was employed with oversampling of underrepresented groups. The 1993 NHIS respondent sample included data from 109,671 persons in 43,007 households selected from around the U.S. In all, a national probability sample of 14,000 women was drawn from the 1993 NHIS. This sampling methodology, used in conjunction with the sampling weights provided by the NSFG (see statistics section below), produced a nationally representative sample. Exclusions from the NSFG included not being born between April 1, 1950 – March 31, 1980, failing to respond or not complete the interview, parental consent not given if ages 15–17, leaving 10,847 women who completed the NSFG interview. Twenty-three women were excluded from this study due to missing asthma/hay fever data. The 1995 NSFG is the only one to include questions related to asthma and/or hay fever. Between January 1995 and October 1995 in-home interviews (90 minute) were conducted providing pregnancy and asthma/hay fever data for 10,824 women 15 to 44 years of age (Table 1).

**Table 1:** NSFG 1995 Respondent Sample with Representation of U.S. Population of Women (15–44 years old).

Characteristic	Range	NSFG Sample		US Population Represented	
		Mean	SD	Mean	SEM
**Age at Interview (years)**	14–45	30.59	8.31	30.09	0.091
Income (% Poverty Index)*	12–998	315.35	219.59	331.06	2.91
Number of Male Partners*	0–21	4.9278	5.35	4.7953	0.07225
**Number of Pregnancies**	0–15	1.97	1.85	1.77	0.024
**Characteristic**	**N**	**Percent**		**Frequency**	**Frequency SE**
**Total**	10,824	100.00%		60,042,017.62	1,236,495.57
**Asthma and/or Hay Fever**					
Hay Fever Only	1,339	12.30%		7,410,212.758	257,315.408
Asthma Only	584	5.40%		3,269,640.363	156,235.741
Both	540	5.00%		3,015,416.326	162,835.575
Neither	8,361	77.20%		4,6346,748.17	1,000,351.235
**Race**					
Black	2,511	14.00%		8,445,847.71	385,472.47
White	7,726	80.00%		47,836,294.18	1,087,051.30
Other	587	6.00%		3,759,875.73	297,244.69
**Hispanic Ethnicity**	1,549	11.00%		6,686,275.83	438,992.52
**Marital Status**					
Married	5,290	49.40%		29,669,734.41	677,835.715
Previously Married	1,552	13.10%		7,844,111.637	277,260.887
Never Married	3,982	37.50%		22,528,171.57	620,086.081
**Geographic Region**					
Northeast	2,028	19.10%		11,487,883	536,788.971
Midwest	2,582	24.10%		14,459,014.4	520,912.278
South	3,742	33.60%		20,169,863.1	549,600.568
West	2,472	23.20%		13,925,257.12	792,537.716
High School Diploma/GED**	8,532	88.00%		47,583,004.97	972,455.74
**Pelvic Inflammatory Disease**	903	7.60%		4,558,407.43	198,558.30
**Diabetes (non-gestational)**	186	2.00%		994,546.27	85,749.50
**Hypertension**	891	7.0%		4,377,586.23	196,425.32
**BMI Category**					
Underweight	497	6.00		3,031,351.29	161,358.98
Normal Weight	6,808	63.00%		40,216,338.63	899,488.38
Overweight	2,075	19.00%		10,116,057.66	292,938.86
Obese	1,444	13.00%		6,678,270.04	202,392.44
**Smoker (≥100 Lifetime Cigarettes)**	4,428	42.00%		25,010,736.61	629,665.50
**Had at Least 1 Pregnancy**	7,759	66.80%		40,095,621.69	899,739.68
**Number of Times Pregnant**					
	**N**	**Percent**			
0	3,079	33.30%		20,048,297.45	552,895.60
1	1,777	16.40%		9,893,230.44	314,673.16
2	2,292	20.30%		12,191,434.53	326,874.10
3	1,702	14.20%		8,551,352.35	285,268.12
4	1,015	8.30%		4,974,282.69	223,056.42
5	507	3.90%		2,323,131.31	121,537.23
6	236	1.90%		1,136,489.11	90,756.84
7	123	0.90%		536,492.65	53,971.42
8	46	0.30%		192,817.30	33,335.16
9	28	0.20%		148,972.52	29,542.86
10	17	0.10%		74,971.45	24,476.70
11	6	0.00%		25,902.09	11,016.81
12	4	0.00%		20,339.39	10,664.98
13	3	0.00%		12,304.49	7,941.76
14	2	0.00%		9,242.17	6,558.79
15	1	0.00%		4,659.22	4,659.22
**Total Lifetime Pregnancies**	21,325			106,538,565.37	*5,537,931.46*

^†^Estimates of population represented based on sampling scheme and weighting used in NSFG 1995^11^.

*Winsorized to 998 for income above 998% of poverty index and 21 for >=21 partners;

**General Equivalency Diploma.

### Study variables

#### Pregnancy outcomes

The history of number of previous pregnancies, live births, spontaneous pregnancy losses (ectopic or tubal pregnancy, miscarriage, stillbirth), and induced abortions are obtained for each woman during the interview. Fertility is operationally defined as the number of previous pregnancies. Fecundity is operationally defined as the number of previous live births. Impaired fecundity is operationally defined as the number of previous spontaneous pregnancy losses. Number of pregnancies was also used as a predictor of impaired fecundity.

#### Lifetime history of asthma and/or hay fever

Lifetime histories of asthma and hay fever are based on responses to questions included in the NHIS that were added to the 1995 NSFG: “Has a doctor or other medical care provider ever told you that you had asthma (yes/no)”/”Has a doctor or other…….ever told you that you had hay fever (yes/no)”^17^. The responses are operationalized into four mutually exclusive categories: asthma only, hay fever only, asthma and hay fever, and neither asthma nor hay fever using three dummy variables with neither asthma nor hay fever as the reference group. The phenotypes, asthma and hay fever and hay fever only, are categorized differently from asthma only based on their significant association with allergen skin test positivity/sensitization^18^, especially during the early reproductive years (≤24 years old), whereas asthma only has a significantly lower association^19^. Self-reported histories of doctor diagnosed hay fever are also highly associated with aeroallergen skin test positivity, whereas self-reported histories of doctor diagnosed asthma are not^19^.

#### Lifetime history of smoking

The response to the question “In your entire life, have you smoked at least 100 cigarettes? (yes/no)” was used to differentiate lifetime nonsmokers (<100) from smokers (>100)^17^.

#### Demographics and health history

The demographic variables obtained included: age at interview, race and Hispanic origin, marital status, region of residence, high school graduation/general equivalency diploma (GED), and income defined as percent federal poverty index at time of interview. The health variables included the biological variables of history of pelvic inflammatory disease (PID), history of diabetes, history of high blood pressure, and Body Mass Index (BMI) classified as underweight (<18.5 kg/m^2^), normal weight (18.5–24.9 kg/m^2^), overweight (25–29.9 kg/m^2^), and obese (>/=30 kg/m^2^)^17^. The health behavior variable of number of male partners since puberty was also obtained.

#### Statistics

Initial descriptive statistics were used to characterize the sample. Because the average age of first heterosexual vaginal intercourse is 15^20^, the number of years of a woman’s potential to become pregnant is calculated by subtracting 15 years from the age at interview. This is akin to mean centering to ensure estimates do not go beyond applicable age ranges. The number of pregnancies per year exposed is calculated for each woman. This rate is used as the fertility outcome. The number of live births per year exposed is calculated for each woman. This rate is the fecundity outcome. The number of spontaneous pregnancy losses per year exposed is calculated for each woman. This rate is used as the impaired fecundity outcome.

Demographic and health variables were added to the analyses as covariates/confounders. Confounders include smoking, age, BMI, race/ethnicity, percent federal poverty index, high school education/GED, and region of residence. Smoking increases the risk of asthma only, decreases the risk of hay fever only^21^ while increasing the risk of pregnancy loss^22^. Age impacts the ability to both become pregnant and maintain pregnancy while also influencing the risk of asthma in women^23^. Obesity increases the risk in women of asthma only^15^ as well as pregnancy loss^24^. As there is a nonlinear relationship between BMI and pregnancy outcomes, BMI categories were included as covariates with normal weight as the reference group. Race/ethnicity is associated with increased asthma prevalence in blacks, increased prevalence in whites of hay fever only^21^ as well as influencing fertility and fecundity^25^. Percent federal poverty index is related to risk of asthma^21^ as well as fertility and fecundity^26^. High school graduation/GED status is related to diagnosis of asthma^27^ as well as risk of pregnancy loss^15^. Region of residence influences risk of asthma^21^ as well as fecundity^28^. Covariates include number of male partners, marital status, PID, hypertension, and diabetes. Number of male partners and marital status are associated with fertility. Marital status^15^, PID^15^, hypertension^29^, and diabetes^30^ are associated with the risk of pregnancy loss.

Poisson regression was used to examine the contribution of asthma and/or hay fever to fertility after controlling for covariates/confounders. Covariates and confounders in the analysis included: smoking, age, race/ethnicity, marital status, region, high school education/GED, income as % poverty index, history of PID, history of hypertension, history of diabetes, BMI, and number of male partners. The potential moderating effect of smoking on the relationship of asthma to fertility was examined by including interaction terms between smoking and asthma/hay fever phenotype into this analysis.

Stratified multivariable Poisson regression analysis was conducted according to smoking status: non-smokers (smoked less than 100 cigarettes in the lifetime) and smokers (smoked 100 or more cigarettes in the lifetime). The analysis of women nonsmokers provided an opportunity to evaluate the relation between asthma/hay fever phenotypes and fertility not confounded by smoking. This analysis included all covariates/confounders from the previous analysis with the exception of the smoking-related variables. The number of smokers/nonsmokers in each asthma/hay fever phenotype ranged between 244 to 775.

Another set of Poisson regression analysis was used to evaluate the contributions of asthma and or hay fever to impaired fecundity. The analysis included only women who had at least one pregnancy that did not end in induced abortion. Initial analyses included examination of the contribution of fertility and of asthma and/or hay fever to impaired fecundity. These were followed by models that included demographic, health, and behavior covariates in addition to these variables. For these analyses, number of pregnancies was a key predictor along with asthma/hay fever phenotype. The covariates/confounders in this set of analyses included: smoking, age, race/ethnicity, marital status, region, high school education/GED, income as % poverty index, history of PID, history of hypertension, history of diabetes, BMI, and number of male partners.

The potential moderating role of asthma only in the relationship between fertility and impaired fecundity was examined in Poisson regression models by adding an interaction term between asthma only (dichotomous) and number of pregnancies per woman year to Poisson regression with asthma only and number of pregnancies as predictors. The graphs illustrate the interactions of fertility with asthma-hay fever status and with smoking status as predictors of impaired fecundity. These analyses controlled for the covariates and confounders included above. These graphs include estimates of pregnancy losses for 70 and 170 pregnancies per 1,000 woman-years, the 25^th^ (NP25) and 75^th^ percentiles (NP75) respectively.

STATA version 12 (Stata Corp., College Station, TX) statistical software program svy was used for multivariable analyses. It computes accurate variances that account for the stratified cluster sampling and oversampling of some subgroups in the sampling design of the NSFG. The NSFG data set variable PANEL was included as the cluster variable, COL_STR as the strata variable, and POST_WT as the sampling weight in all analyses. Estimated marginal means were compared using t-tests with Holm’s sequential Bonferroni corrections for multiple comparisons.

#### Ethics

The 1995 NSFG survey was carried out in accordance with relevant ethical guidelines and regulations. The 1995 NSFG survey protocols were reviewed and approved by the National Center for Health Statistics (NCHS) Institutional Review Board/NCHS Research Ethics Review Board. Informed consent was obtained from all subjects or if subjects were under 18 years old from a parent or legal guardian.

## Results

### Demographics

A total of 10,824 women in the NSFG represents approximately 60 million US women aged 15–44 years. Of these 66.8% have at least one pregnancy by the time of their interview experiencing a total of 21,325 pregnancies, with the number of pregnancies ranging from 0 to 15 per woman. Demographic, health, and behavioral data for the sample and the population they represent are presented in Table 1.

### Fertility (number of pregnancies per woman-year)

Women experienced a mean fertility rate of 106.14 pregnancies per 1000 woman-years (95% CI = 103.0, 109.3). In Poisson regression with all aforementioned covariates, asthma only is a significant independent predictor of more pregnancies compared to women with neither asthma nor hay fever. No significant differences in number of pregnancies are observed in women with asthma and hay fever or hay fever only compared to women with neither asthma nor hay fever. (Table 2). Younger age, lower income, and less education (no high school diploma/GED) independently predict higher number of pregnancies per fertile year after controlling for asthma/hay fever and all other covariates. Being married or previously married (compared with never married), Hispanic or non-Hispanic black (compared with non-Hispanic white), being overweight (compared with normal weight), smoking (≥lifetime 100 cigarettes), and having more male partners since puberty also independently predict higher number of pregnancies after controlling for all other covariates/confounders (Table 2). Smoking is also associated with higher fertility (Table 2). Based on these findings, the interactions between smoking and the asthma and hay fever variables were added to the Poisson regression model.

**Table 2:** Fertility in All Women: Results of Poisson regression examining the independent contributions of asthma/hay fever categories and other predictors to number of pregnancies per woman-year in women aged 15–44 years in the NSFG (*N = 9,284).

Characteristic	Coeff.	Std. Error	t	p	95% Confidence Interval
**Asthma and/or Hay Fever**					
Asthma Only	0.1196	0.0482	2.48	0.014	0.0245, 0.2148
Hay Fever Only	0.0053	0.0320	0.17	0.868	−0.0578, 0.0685
Asthma & Hay Fever	0.0203	0.0452	0.45	0.654	−0.0688, 0.1093
Neither	(ref)				
**Age**	−0.0204	0.0016	−12.9	<0.001	−0.0235, −0.0173
**Race/Ethnicity**					
Hispanic	0.1625	0.0311	5.23	<0.001	0.1012, 0.223
Non-Hispanic Black	0.3935	0.0276	14.28	<0.001	0.3392, 0.4479
Non-Hispanic Others	0.0641	0.054	1.19	0.234	−0.0417, 0.1700
Non-Hispanic White	(ref)				
**Marital Status**					
Married	0.8643	0.0386	22.41	<0.001	0.7882, 0.9403
Previously Married	0.6572	0.0440	14.95	<0.001	0.5704, 0.7439
Never Married	(ref)				
**Region**					
North East	0.0077	0.0319	0.24	0.810	−0.0553, 0.0706
Midwest	−0.0407	0.0354	−1.15	0.251	−0.1105, 0.0290
South	−0.1515	0.0289	−5.24	<0.001	−0.2086, −0.0945
West	(ref)				
**High School Grad or GED**	−0.3651	0.0275	−13.28	<0.001	−0.4193, −0.3108
**% Poverty Index**	−0.0012	0.0011	−20.01	<0.001	−0.0014, −0.0011
**PID**	0.0602	0.0321	1.87	0.063	−0.0032, 0.1236
**Diabetes**	−0.1410	0.0902	−1.56	0.120	−0.3190, 0.0370
**Hypertension**	−0.0708	0.0386	−1.84	0.068	−0.1469, 0.0053
**BMI Category**					
Underweight	<0.0015	0.0485	0.01	0.992	−0.0951, 0.0961
Overweight	0.0889	0.0205	4.33	<0.001	0.0484, 0.1294
Obese	0.0504	0.0262	1.92	0.056	−0.0014, 0.1021
Normal Weight	(ref)				
**Smoker**	0.1265	0.0191	6.61	<0.001	0.0887, 0.1642
**Number Male Partners**	0.0226	0.0018	12.78	<0.001	0.0191, 0.0261
Constant	−1.709	0.0684	−24.99	<0.001	−1.8439, −1.5741

GED general equivalency diploma; ref reference group.

*N reduced due to missing data.

### Fertility in smokers/nonsmokers with asthma/hay fever phenotypes

**Figure 1:**
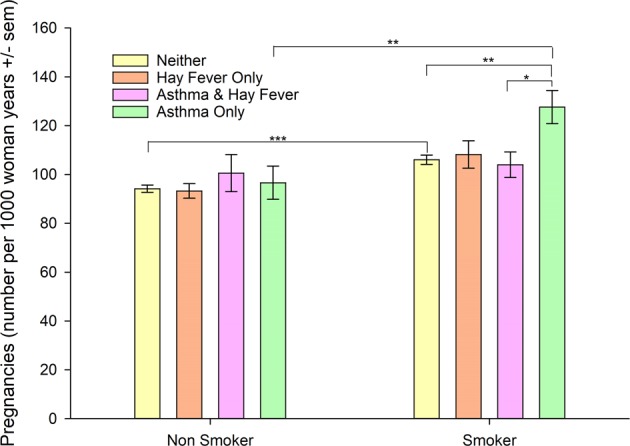
Estimated fertility (number of pregnancies per 1,000 woman-years) according to asthma/hay fever diagnosis and smoking status controlling for age, race/ethnicity, marital status, region, education, income, pelvic inflammatory disease, diabetes, hypertension, BMI, and number of male partners from the NSFG (N = 9,284). Estimates based on complex samples analysis in Table 2 adjusted for multiple comparisons, *p < 0.05, **p < 0.01, ***p < 0.001.

The significant interaction between asthma only and smoking (β = 0.1590, t = 1.97, 95% CI = 0.0000, 0.3180, p = 0.05) indicates that the presence of both is associated with increases in the number of pregnancies (Table 2). None of the other asthma/hay fever interactions with smoking were significant. Estimates of fertility (number of pregnancies per 1,000 woman-years) in smokers and non-smokers according to asthma/hay fever status and smoking status, controlling for all other covariates shown in Table 2, are included in Fig. 1.

In smokers, there is no significant difference in fertility between women with hay fever only compared to women with neither asthma nor hay fever. Among non-smokers there is also no significant difference in fertility in women with hay fever only or asthma and hay fever compared to women nonsmokers with neither asthma nor hay fever (Fig. 1). The pattern of the significance of the relationships of the other covariates from age to number of male partners (Table 2) to number of pregnancies did not change.

The moderation effect of smoking on the relationship of asthma/hay fever status to number of pregnancies is evaluated using stratified multivariable Poisson regression analyses performed on non-smokers (Table 3) and smokers (Table 4). Among women who smoked fewer than 100 cigarettes in their lifetimes (non-smokers), asthma only is not a significant independent predictor of the number of pregnancies per woman year compared to women with neither asthma nor hay fever (Table 3). The relationships of the other covariates to number of pregnancies remains relatively unchanged except for hypertension which predicts lower numbers of pregnancies (Table 3).

**Table 3:** Fertility in Women Nonsmokers: Results of Poisson regression examining the independent contributions of asthma/hay fever categories and other predictors to number of pregnancies per woman-year in nonsmokers (<100 lifetime cigarettes) aged 15–44 years in the NSFG (N = 5,197).

Characteristic	Coeff.	Std. Error	t	p	95% Confidence Interval
**Asthma and/or Hay Fever**					
Asthma Only	0.0324	0.0665	0.49	0.626	−0.0988, 0.1637
Hay Fever Only	−0.0194	0.0345	−0.56	0.576	−0.08751, 0.0487
Asthma & Hay Fever	0.0684	0.0762	0.90	0.370	−0.0819, 0.2187
Neither	(ref)				
**Age**	−0.0113	0.0019	−5.85	<0.001	−0.0151, −0.0075
**Race/Ethnicity**					
Hispanic	0.1157	0.0420	2.75	0.007	0.0328, 0.1986
Non-Hispanic Black	0.4673	0.0355	13.17	<0.001	0.3973, 0.5373
Non-Hispanic Others	0.0101	0.0656	0.15	0.877	−0.1194, 0.1396
Non-Hispanic White	(ref)				
**Marital Status**					
Married	1.026	0.0509	20.17	<0.001	0.9257, 1.1265
Previously Married	0.6865	0.0568	12.08	<0.001	0.5744, 0.7986
Never Married	(ref)				
**Region**					
North East	0.0309	0.0433	0.71	0.476	−0.0545, 0.1162
Midwest	−0.0230	0.0470	−0.49	0.625	−0.1158, 0.0697
South	−0.1391	0.0431	−3.23	0.001	−0.2240, −0.0541
West	(ref)				
**High School Grad or GED**	−0.3922	0.0385	−10.19	<0.001	−0.4682, −0.3163
**% Poverty Index**	−0.0015	0.0011	−16.67	<0.001	−0.0017, −0.0013
**PID**	0.0939	0.0491	1.91	0.058	−0.0031, 0.1908
**Diabetes**	−0.0443	0.1525	−0.29	0.772	−0.3452, 0.2566
**Hypertension**	−0.1143	0.0449	−2.54	0.012	−0.2029, −0.0256
**BMI Category**					
Underweight	−0.0817	0.0761	−1.07	0.285	−0.2319, 0.0685
Overweight	0.0659	0.0315	2.09	0.038	0.0037, 0.1281
Obese	0.0590	0.0373	1.58	0.115	−0.0145, 0.1326
Normal weight	(ref)				
**Number Male Partners**	0.0255	0.0031	8.34	<0.001	0.0195, 0.0315
Constant	−2.0227	0.0928	−21.79	<0.001	−2.2059, −1.8396

GED general equivalency diploma; ref reference group.

**Table 4:** Fertility in Women Smokers: Results of Poisson regression examining the independent contributions of asthma/hay fever categories and other predictors to number of pregnancies per woman-year in smokers (≥100 lifetime cigarettes) aged 15–44 years in the NSFG (N = 4,037).

Characteristic	Coeff.	Std. Error	t	p	95% Confidence Interval
**Asthma and/or Hay Fever**					
Asthma Only	0.1934	0.0563	3.44	0.001	0.0824, 0.3044
Hay Fever Only	0.0228	0.0504	0.45	0.652	−0.0767, 0.1222
Asthma & Hay Fever	−0.0303	0.0521	−0.58	0.562	−0.1331, 0.0726
Neither	(ref)				
**Age**	−0.0292	0.0024	−12.41	<0.001	−0.0339, −0.0246
**Race/Ethnicity**					
Hispanic	0.2086	0.0410	5.08	<0.001	0.1277, 0.2896
Non-Hispanic Black	0.3258	0.0409	7.97	<0.001	0.2451, 0.4065
Non-Hispanic Others	0.1516	0.0889	1.71	0.090	−0.0238, 0.3270
Non-Hispanic White	(ref)				
**Marital Status**					
Married	0.6790	0.0504	13.47	<0.001	0.5796, 0.7785
Previously Married	0.5734	0.0592	9.68	<0.001	0.4565, 0.6903
Never Married	(ref)				
**Region**					
North East	−0.0240	0.0402	−0.60	0.550	−0.1034, 0.0553
Midwest	−0.0671	0.0453	−1.48	0.141	−0.1565, 0.0224
South	−0.1715	0.0367	−4.68	<0.001	−0.2438, −0.0991
West	(ref)				
**High School Grad or GED**	−0.3506	0.0373	−9.40	<0.001	−0.4242, −0.2770
**% Poverty Index**	−0.0010	0.0000	−12.50	<0.001	−0.0011, −0.0008
**PID**	0.0344	0.0439	0.79	0.443	−0.0521, 0.1210
**Diabetes**	−0.1621	0.0836	−1.94	0.054	−0.3271, 0.0029
**Hypertension**	−0.0341	0.0565	−0.60	0.547	−0.1455, 0.0773
**BMI Category**					
Underweight	0.0737	0.0657	1.12	0.263	−0.0559, 0.2033
Overweight	0.1071	0.0279	3.84	<0.001	0.0521, 0.1623
Obese	0.0197	0.0378	0.46	0.645	−0.0572, 0.0921
Normal Weight	(ref)				
**Number Male Partners**	0.0175	0.0021	9.10	<0.001	0.0155, 0.0240
Constant	−1.2141	0.0884	−13.73	<0.001	−1.389, −1.040

GED general equivalency diploma; ref reference group.

Among smokers, asthma only is a significant independent predictor of increased number of pregnancies per woman year compared to women with neither asthma nor hay fever (Table 4). In smokers, hay fever only and asthma and hay fever are not significant independent predictors of the number of pregnancies compared to women with neither asthma nor hay fever (Table 4). The relationships of the other covariates to number of pregnancies in smokers remains relatively unchanged (Table 4) compared to the analysis of all women (Table 2).

### Fecundity (number of live births per woman-year)/Impaired Fecundity (number of spontaneous pregnancy losses per woman-year)

Fecundity and impaired fecundity were examined using pregnancies that did not end in induced abortions. Pregnancy losses and number of pregnancies (excluding abortion) are available from 7,658 respondents who had been pregnant at least once, representing approximately 39,413,900 women. Women experienced 135.0 pregnancies per 1000 woman-years (95% CI = 132.1, 138.1) that did not end in induced abortion. Among these women were 401 respondents, representing approximately 2.5 million women, who reported zero pregnancies that did not end in abortion. The pregnancies resulted in 110.33 live births per 1000 woman-years and 25.0 spontaneous pregnancy losses per 1000 woman-years (95% CI = 23.1, 26.3) occurring in 18.5% of pregnancies reported. Five thousand four hundred eighty-five (5,485) respondents (71.6%), representing approximately 28 million women, did not experience spontaneous pregnancy losses.

### Asthma phenotypes and impaired fecundity

Asthma only is analyzed as a contributor to the relationship of number of pregnancies and pregnancy losses. In bivariate Poisson regression analyses, asthma only, operationalized as a dichotomous variable, predicts both higher number of pregnancies (β = 0.2957, t = 6.76, 95% CI = 0.2094, 0.3819, p < 0.001) and higher pregnancy losses (β = 0.6807, t = 5.37, 95% CI = 0.4308, 0.9306, p < 0.001).

The role of asthma only as a moderator of the relationship between pregnancy and pregnancy losses is examined using Poisson regression. With the number of pregnancies per woman-year, asthma only and their interaction as predictors of spontaneous losses per woman-year, their interaction is a significant independent predictor (β = 0.9330, t = 2.03, 95% CI = 0.0283, 1.8380, p = 0.043). The greater the number of pregnancies, the greater the contribution of asthma only to pregnancy losses.

### Smoking and impaired fecundity

The role of smoking as a moderator of the relationship between pregnancies and pregnancy losses is examined in Poisson regression. With the number of pregnancies per woman-year, smoking and their interaction as predictors of pregnancy losses per woman-year, their interaction is a significant independent predictor (β = −1.5709, t = −3.42, 95% CI = −2.4760, −0.6658, p = 0.001). The greater the number of pregnancies, the lesser the relationship of smoking to pregnancy losses. The relationship of number of pregnancies to the number of pregnancy losses is lower in smokers than in non-smokers.

### Rates of pregnancy losses in smokers/nonsmokers

**Figure 2:**
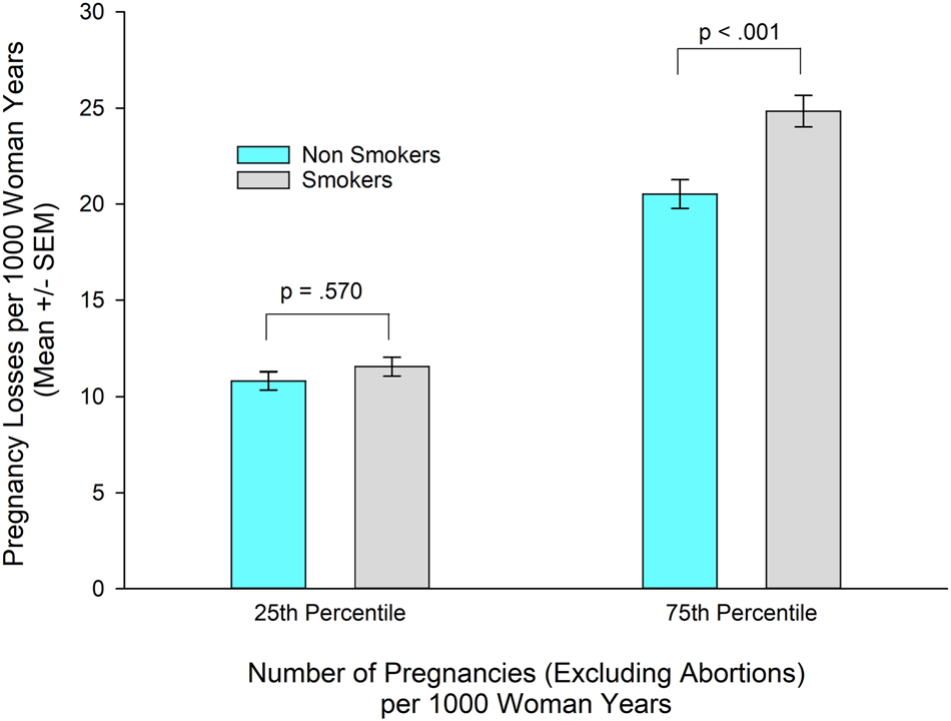
Estimated impaired fecundity (number of pregnancy losses per 1000 woman-years) according to smoking status and number of pregnancies per thousand woman years, controlling for age, race/ethnicity, marital status, region, education, income, pelvic inflammatory disease, diabetes, hypertension, BMI, number of male partners and number of pregnancies as well as the interaction between asthma/hay fever status and number of pregnancies from the NSFG (N = 9,284). Estimates based on complex samples analysis in Table 5 adjusted for multiple comparisons.

The association of the interaction of smoking and number of pregnancies per 1,000 woman-years with spontaneous pregnancy losses per 1,000 woman-years is illustrated in Fig. 2. The estimate of the rate of pregnancy losses among smokers at NP75 is significantly higher than among nonsmokers after controlling for all covariates/confounders. In contrast, there is no significant difference in rates of pregnancy losses of smokers compared to nonsmokers at NP25.

### Asthma phenotypes/smoking and impaired fecundity

A multivariable Poisson regression analysis of number of spontaneous pregnancy losses that includes the four categories of asthma and/or hay fever and all the previous covariates/confounders plus interaction terms for asthma/hay fever categories with number of pregnancies and for smoking with number of pregnancies is present in Table 5. The interaction of asthma only with number of pregnancies per woman year and the interaction of smoking with number of pregnancies, each are independent predictors of greater number of pregnancy losses per woman-year. None of the other asthma/hay fever categories had significant interactions with number of pregnancies as predictors of number of pregnancy losses. Other significant predictors of pregnancy losses are the number of pregnancies, being older, never being married, having a high school diploma, higher income, PID, and more male partners (Table 5). These findings suggest independent roles of asthma only and smoking in the process leading from number of pregnancies to pregnancy losses.

**Table 5:** Association of fertility with impaired fecundity: Results of Poisson regression examining the independent contributions of pregnancies per woman-year (excluding abortion), asthma only, smoking, the interactions of asthma and of smoking with number of pregnancies and other predictors to number of spontaneous pregnancy losses per woman-year (excluding abortion) in women aged 15–44 years in the NSFG (*N = 7,239).

Characteristic	Coeff.	Std. Error	t	p	95% Confidence Interval
**Number of Pregnancies (P)**	6.4854	0.2780	23.33	<0.001	5.9369, 7.0340
**Asthma and/or Hay Fever**					
Asthma Only	0.4240	0.1452	2.92	0.004	0.1375, 0.7105
Hay Fever Only	0.0229	0.1274	0.18	0.858	−0.2286, 0.2743
Asthma & Hay Fever	0.3496	0.1203	2.91	0.004	0.1123, 0.5869
Neither	(ref)				
**Smoke**	−0.0253	0.0688	−0.37	0.714	−0.1611, 0.1105
**Asthma and/or Hay Fever X**^†^ **P**					
Asthma Only X P	−1.1133	0.3811	−2.92	0.004	−1.8651, −0.3615
Hay Fever Only X P	−0.0954	0.6940	−0.14	0.891	−1.4646, 1.2738
Asthma & Hay Fever X P	−0.9257	0.4924	−1.88	0.062	−1.8970, 0.0456
**Smoke X P**	1.3020	0.3130	4.16	<0.001	0.6846, 1.9194
**Age**	0.0228	0.0046	4.96	<0.001	0.0137, 0.0318
**Race/Ethnicity**					
Hispanic	−0.0811	0.0711	−1.14	0.255	−0.2213, 0.0591
Non-Hispanic Black	−0.1286	0.0791	−1.62	0.106	−0.2846, 0.0276
Non-Hispanic Others	−0.0617	0.1444	−0.43	0.670	−0.3465, 0.2231
Non-Hispanic White	(ref)				
**Marital Status**					
Married	−0.2169	0.0872	−2.49	0.014	−0.3890, −0.0449
Previously Married	−0.2221	0.0967	−2.30	0.023	−0.4128, −0.0313
Never Married	(ref)				
**Region**					
North East	−0.0076	0.0708	−0.11	0.915	−0.1473, 0.1322
Midwest	−0.1018	0.0847	−1.20	0.231	−0.2689, 0.0654
South	0.0192	0.0731	0.26	0.793	−0.1250, 0.1634
West	(ref)				
**High School Grad or GED**	0.2237	0.0652	3.43	<0.001	0.0951, 0.3522
**% Poverty Index**	0.0010	0.0001	8.40	<0.001	0.0007, 0.0012
**PID**	0.3044	0.0712	4.27	<0.001	0.1639, 0.4449
**Diabetes**	−0.0479	0.2445	−0.20	0.845	−0.5302, 0.4344
**Hypertension**	−0.0058	0.0930	−0.06	0.951	−0.1891, 0.1776
**BMI Category**					
Underweight	0.0904	0.0993	0.91	0.364	−0.1055, 0.2863
Overweight	0.0352	0.0591	0.60	0.552	−0.0814, 0.1517
Obese	0.1031	0.0828	1.25	0.214	−0.0601, 0.2664
**Number Male Partners**	0.0088	0.0045	1.98	0.050	0.0000, 0.0768
Constant	−6.1463	0.2041	−30.11	<0.001	−6.5490, −5.7436

X^†^ = interaction; GED general equivalency diploma; ref reference group.

*N reduced due to missing data.

### Rates of pregnancy losses in asthma/hay fever phenotypes

**Figure 3:**
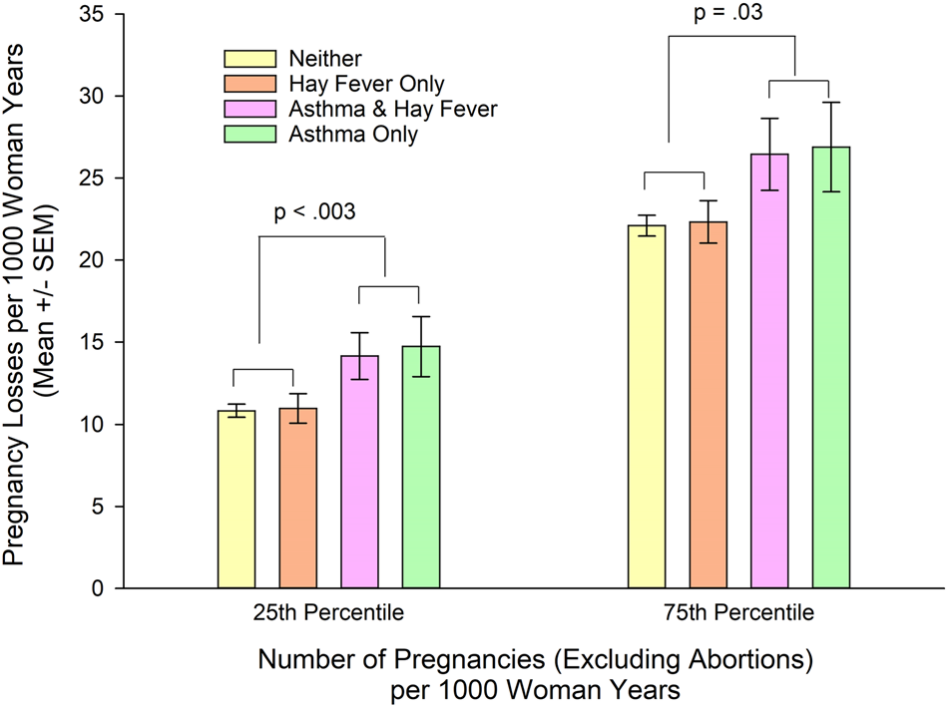
Estimated impaired fecundity (number of pregnancy losses per 1000 woman-years) according to asthma/hay fever status and number of pregnancies per thousand woman years, controlling for age, race/ethnicity, marital status, region, education, income, pelvic inflammatory disease, diabetes, hypertension, BMI, number of male partners, and number of pregnancies as well as the interaction of number of pregnancies with smoking from the NSFG (N = 9,284). Estimates based on complex samples analysis in Table 5 adjusted for multiple comparisons.

The association of the interactions of asthma and/or hay fever and number of pregnancies per 1,000 woman-years with spontaneous pregnancy losses per 1,000 woman-years is illustrated in Fig. 3. At NP75, neither of the estimates of rates of pregnancy losses with asthma only (26.90 ± 2.73) nor of asthma and hay fever (26.45 ± 2.20) are significantly different from the estimate for women with neither asthma nor hay fever [22.10 ± 0.63); t(5924) = 1.84, p = 0.065 and t(5935) = 1.72, p = 0.17, respectively]. In contrast, the estimate of rates of pregnancy losses in women with asthma only at NP25 (14.74 ± 1.81) is significantly higher compared to women with neither [10.84 ± 0.40, t(5924) = 2.38, p = 0.017], whereas the rate in women with asthma and hay fever (14.16 ± 1.41) is not significantly different from women with neither [10.84 ± 0.40; t(5935) = 2.09, p = 0.074]. Although not reaching statistical significance, the rates of spontaneous pregnancy losses for asthma and hay fever are similar to asthma only (Fig. 3). The contribution of number of pregnancies to pregnancy losses depends on asthma (with and without hay fever) [β = 0.9977, t = 2.97, 95% CI = 0.3359, 1.6596, p = 0.003]. After grouping asthma only and asthma and hay fever together, asthma predicts significantly increased rates of spontaneous pregnancy losses compared with women without asthma after controlling for interactions and covariates/confounders. Figure 3 illustrates estimated spontaneous pregnancy losses at NP25 and NP75.

## Discussion

Fertility findings in the current analysis are consistent with U.S. data. The overall fertility rate of 106.14 pregnancies per 1,000 woman-years is similar to that previously reported^31^. Asthma only, smoking, and younger age are predictors of significantly higher fertility (Table 2) consistent with the highest U.S. pregnancy rate (1976–1996) in the age 20–24 cohort^25^. The differing asthma/hay fever phenotypes and their associated endotypes provide an immunologic rationale for the association of smoking and asthma only smokers with increased fertility. Asthma/hay fever phenotypes differ by age and smoking in their association with allergen skin test positivity^19^. During the early reproductive years (≤24 years old), the clinical phenotypes hay fever only, regardless of smoking, and asthma and hay fever in nonsmokers are significantly associated with aeroallergen skin test positivity (number of positive skin tests)^19^. Skin test positivity to either ragweed or rye grass in the U.S. population (<24 years old) increases the odds of hay fever only 160–200% and skin test positivity to *Alternaria* increases the odds of asthma and hay fever 860%^32^. Proteases in ragweed, rye grass, and *Alternaria* aeroallergens can activate a T2 immune response^33^. These clinical phenotypes are atopic, consistent with T2 inflammatory endotypes, respectively termed T2 immune response rhinitis^9,10^ and T2-high asthma^34,35^. T2-high asthma is associated with eosinophilia^36^. The asthma and hay fever phenotype accounts for about 50% of asthma prevalence^37^ in women (Table 1) and has been characterized as being mild to moderate in severity^34^.

In contrast, the prevalence of asthma only (without hay fever) in the early reproductive years as well as during the later reproductive years (25–49 years of age) in nonsmokers has a significantly lower association with aeroallergen skin test positivity^19^ and include endotypes classified as T2-low asthma^37–39^/non-T2 Type 1 (T1) asthma^34,40^. The nonatopic phenotype, asthma only, also accounts for about 50% of asthma^39,41^ and “…may be highly prevalent in mild to moderate asthmatics in the general population”.^42^

Immune deviation from a T2-high asthma to a T2-low asthma/T1 endotype is characterized by biomarkers of neutrophil recruitment e.g., IL-1alpha, IL-6, IL-8^43^, innate immune response dysregulation e.g., IL-23, TNF alpha, interferon, IL-17^44–48^, and includes neutrophilic noneosinophilic asthma^49^. Neutrophilic asthma is significantly increased in smokers with asthma compared to nonsmokers with asthma^50^ and in previous smokers with severe asthma compared to never smokers with severe asthma^51^.

The proinflammatory T2-low asthma endotype has similar biomarker characteristics to the fetal-maternal interface during implantation. Prior to implantation^52,53^ and in the peri-implantation period the fetal-maternal interface is also characterized by immune deviation to a proinflammatory^54^ (IL-1beta, IL-6, LIF, PGE2, CXCL8, IL-17A, TNF) T2-low endotype^55–57^. Insemination, exposure to semen, initiates a short lived neutrophilic inflammatory (IL-1beta, TNF alpha, CxCL1, IL-17A) internal genital response^58–60^. The placental cytokines, IL-1beta, IL-6, TNFalpha, as well as increased PGE2^61,62^, are also associated with early onset (2 weeks after fertilization) pregnancy symptoms e.g., nausea/vomiting^62^. PGE2, a negative regulator of type 2 innate lymphoid cell response^63^, is a mediator of cough variant asthma^64,65^ in whom half studied are nonatopic and nearly two-thirds women^66,67^. Soluble HLA-G is both a biomarker of the T2-low asthma endotype^68^ and a tolerance inducing MHC molecule that facilitates implantation at the fetal-maternal interface^69^. It is found in T2-low severe asthma in whom two-thirds studied were women and fifty percent on oral glucocorticosteroids^68^.

Smoking significantly increases the risk of asthma only in the U.S population^21^ and elicits a systemic proinflammatory response^70–72^ including IL-1beta and IL-17 shifted cytokine profiles^73^. Thus deviation towards a T2-low endotype in smokers and smokers with asthma only may be permissive for embryo implantation in the early and prime reproductive years underlying the significantly positive interaction observed between smoking and asthma only (see Results) that predicts an even greater increase in fertility for asthma only smokers compared to other smokers (Fig. 1) and significantly increased fertility in smokers compared to nonsmokers. In healthy women, there is a balance of T2-high and T2-low endotypes during embryo implantation^74^.

Predictors of fertility are consistent with previous studies. Proximate behavioral determinants of fertility include sexual exposure e.g., number of partners, marital/cohabiting status, and contraception^75^. Increased fertility of married women, of non-Hispanic blacks, of Hispanics compared to non-Hispanic whites, lower income, and educational attainment (Tables 2–4) have been reported^26,76^. In this respect, sexually active Hispanic, non-Hispanic black women, and women with lower education or lower income report lower use of contraceptives^77^. Differences in fertility associated with overweight/obesity may be due to behavioral factors as obese women utilize sterilization as a method of contraception more often^78^, have decreased marriage rates^79^, and a significantly higher risk of lifetime childlessness than overweight women^80^. Smoking is associated with risky sexual/health behaviors^81^, sexually transmitted diseases^82–85^, number of sexual partners^85,86^, and failure to use contraception^86^.

The covariates/confounders significantly associated with spontaneous pregnancy losses in the current study (Table 5) are consistent with those in previous reports. The percent of women (28.4%) experiencing a spontaneous pregnancy loss (see Results: Fecundity) is similar to the 28% recently reported^87^. Predictors of spontaneous pregnancy losses previously reported include increased age^15,88^, history of PID^15,89,90^, high school graduate^15^ (or equivalent)^16^/higher education^88^, never having married^15,88^, higher income^16,88^, prior pregnancies^88^, smoking^22^, and number of male partners^91^.

The number of pregnancies is a significant independent predictor of pregnancy losses (Table 5). Women with pregnancy losses i.e., impaired fecundity are less likely to use contraceptives and more likely to have unintended pregnancies^92^. In interviews of women with impaired fecundity who had unintended pregnancies, over 60% of those who became pregnant believe they could not become pregnant or didn’t mind becoming pregnant^92^. As ovulation resumes 20 days (median) after a spontaneous pregnancy loss and before the next menses, women may not have the necessary reproductive health information to be aware they may be able to conceive shortly after miscarriage^93,94^. Thus women with impaired fecundity may not be prepared to use contraception sufficiently early after a miscarriage^95^ or have information pertinent to the most effective contraceptive methods^96^.

This study which analyzes history of previous pregnancy losses contrasts with our previous analyses of pregnancy loss based on the most recent singleton pregnancy. In that study, women (including smokers) with asthma only, but not women with asthma and hay fever, experienced an 80% increased odds of spontaneous pregnancy loss compared to those who had neither asthma nor hay fever^15^. Nonsmokers with either asthma only or asthma and hay fever, in that study, also did not have increased pregnancy loss^15^. Similarly, in this study, when individual phenotypes are analyzed, only women with asthma only at the lower number of previous pregnancies (NP25) have significantly increased rates of pregnancy losses (see Results: Rates of pregnancy losses in asthma/hay fever phenotypes). Common to both studies, smoking is an independent predictor of increased fertility (Table 2) in this study and a mediator with asthma of spontaneous pregnancy loss based on the most recent singleton pregnancy^15^. This pattern of pregnancy loss in our prior study in which the significant increase in pregnancy loss is observed only in women with asthma only that included smokers is similar to the pattern of fertility in this study in which the significant increase in fertility is observed only in asthma only smokers (Fig. 1) suggesting that the population of women selected based on their most recent singleton pregnancy may be representative of women with the highest fertility.

Increasing age and the moderation of smoking by number of pregnancies predict impaired fecundity (Table 5). This finding may be related to age and smoking associated endotypic changes in asthma/hay fever phenotypes. During the later reproductive years (>24 years old), the association of allergen skin test positivity with the prevalence of hay fever only increases, regardless of smoking^19^. In contrast, there is decreased association of allergen skin test positivity with the prevalence of asthma only as well as asthma and hay fever in nonsmokers after age 24 years compared to earlier reproductive years^19^. These findings suggest greater importance of nonallergic asthma etiologies as contributors to impaired fecundity in women with asthma and hay fever as they age as well as with asthma only.

Examples of nonallergic etiologies of asthma/asthma exacerbations, in addition to smoking, that are also associated with spontaneous pregnancy loss include infectious agents^97,98^, outdoor air pollution^99–101^, and indoor air pollution^102,103^. Indoor air contaminated by phthalates, ubiquitous semi-volatile endocrine-disrupting chemicals e.g., di(2-ethylhexyl) phthalate used as plasticizers in polyvinyl chloride plastics and ingredients in personal care products/cosmetics^104,105^, result in higher phthalate exposure in women^106^ and women of color^107^. Phthalate exposure is associated with an increased risk of endometriosis^108^. Endometriosis also increases the risk of spontaneous pregnancy loss^109^ and women with asthma also have an increased risk of endometriosis^110^. The sexually dimorphic increased incidence of nonallergic asthma in young women following puberty^111^ has been attributed to fluctuations in endogenous sex hormones as menarche, menstrual irregularity, pregnancy, and menopause as well as exogenous sex hormones (oral contraceptives and hormone replacement therapy) influence asthma exacerbations/remissions^112^. Phthalates in addition to being studied in association with spontaneous pregnancy loss^103^ have also been detected in intrafollicular fluid during oocyte retrieval for fertility treatment^113^. Intrafollicular and serum phthalate levels are associated with alterations in levels of serum reproductive hormones e.g., decreased anti-Mullerian hormone^114^ as well as follicular ovarian reserve hormones^113^. Decreased anti-Mullerian hormone is associated with increased risk of spontaneous pregnancy loss^115^.

The incidence of nonallergic asthma remains significantly higher in women than men throughout their reproductive years (>20 years old), with both higher incidence and prevalence of nonallergic asthma observed in the later reproductive years (>35 years old)^116^. Nonallergic asthma in young adults is also significantly increased in women and is associated with decreased allergen sensitization and decreased T2 markers consistent with T2-low asthma^117^. Older age, decreased allergen sensitization, and absence of allergic rhinitis are also associated with more severe asthma^118^. Nonallergic asthmatics have increased asthma exacerbations during pregnancy^119,120^. Airway reactivity associated with sputum neutrophilia is increased in asthmatic women during pregnancy^119^.

Although asthma severity is not associated with increased risk of spontaneous pregnancy losses, uncontrolled asthma (emergency room visits/hospitalization/systemic corticosteroids), especially in women >34 years old is^121^. IL-8 is a biomarker of uncontrolled asthma and is associated with increased blood^122^ and bronchoalveolar lavage (BAL) neutrophils^123^, oral/parenteral glucocorticoid unresponsiveness^122^, and reduced pulmonary function^122,123^. Endotypes of treatment resistant severe asthma include neutrophilic T2-low asthma^43,49,124^ e.g., T2-low/Th17-low^43^, T1/Th17^125,126^ which are associated with subclinical bronchial infection, increased IL-8^43^, lower airway dysbiosis^127^, and systemic inflammation^128^.

Immune deviation towards a T2-low asthma endotype in women as they age with asthma and hay fever and with asthma only provides an immunologic rationale for these asthma/hay fever phenotypes being associated with impaired fecundity. Immune deviation away from a T2 endotype^129^ at the fetal-maternal interface after implantation contributes to spontaneous abortion and recurrent miscarriage^130–132^. Thus the nonallergic asthma phenotype, asthma only, which includes T2-low endotypes, might be permissive for embryo implantation facilitating fertility in younger women (Fig. 1), but not support gestation (Fig. 3). As there is decreased allergen skin test positivity associated with the prevalence of asthma and hay fever during the later (>24 years old) reproductive years^19^ and as older age is a significant predictor of impaired fecundity (Table 5), the increased prevalence of nonallergic etiologies, T2-low endotypes, of asthma and hay fever in adult women may account for the increased rate of pregnancy losses in women with asthma and hay fever that is similar to asthma only (Fig. 3).

Severe neutrophilic asthma that skew towards a Th17 mediated immune response is also observed in a subset of asthma patients^133,134^. An imbalance in the ratio/function of Th17 (increased) with respect to Treg (decreased) has been observed in asthma^135,136^ including neutrophilic asthma^137^ as well as in recurrent spontaneous pregnancy loss^138^.

Immune deviation towards a T2-high endotype at the fetal-maternal interface after implantation contributes to maintaining immune tolerance of the semiallogeneic fetus during normal pregnancy^139–141^. Thus hay fever only, a T2-high endotype, is consistent with preserved fecundity in women with hay fever only (Fig. 3).

This study demonstrates the epidemiologic differences associated with fertility and impaired fecundity. Limitations of the 1995 NSFG include self-reports of pregnancies and diagnosed medical conditions, both subject to recall bias. The NSFG data do not include contraceptive use prior to each prior pregnancy, medications used, severity of asthma symptoms, adequacy of asthma treatment, tests of pulmonary function, menstrual irregularity, or polycystic ovary syndrome. Influence of age of menarche and endometriosis are not included in this analysis. The 1995 NSFG did not obtain laboratory samples/biomarkers precluding hormonal or endotype assessment.

Within the broad phenotype/endotype categories discussed, there are complex heterogenous subtypes of asthma^142^ including asthma and chronic obstructive pulmonary disease overlap^143^ as well as subtypes of allergic rhinitis^144^ that will need to be studied in relation to fertility/fecundity. Although subsequent NSFGs have not included questions pertinent to asthma and hay fever, the estimated prevalence of asthma and hay fever from the 1995 NSFG and the other risk factors associated with fertility and/or fecundity observed in this study are consistent with literature cited subsequent to the 1995 NSFG.

## Conclusions

Asthma only is associated with the increased risks of both becoming pregnant and failing to maintain a viable pregnancy. These effects are confounded by increased fertility and pregnancy losses associated with smoking. The 1995 NSFG sharpens the focus on women smokers with asthma only who have both significantly increased fertility and impaired fecundity compared to other smokers. Women who have asthma or smoke require special attention to reproductive health education, family planning, and smoking cessation as well as optimal asthma/medical management^145,146^ of their high risk pregnancies prenatally and throughout gestation.

## Data Availability

The datasets generated during and/or analysed during the current study are available in the NSFG Cycle 5 (1995): Public Use Data Files, Codebooks, and Documentation, [https://www.cdc.gov/nchs/nsfg/nsfg_cycle5.htm].
