# Author Correction: Asthma and/or hay fever as predictors of fertility/impaired fecundity in U.S. women: National Survey of Family Growth

**DOI:** 10.1038/s41598-020-64338-0

**Published:** 2020-04-23

**Authors:** Paul C. Turkeltaub, Richard F. Lockey, Katie Holmes, Erika Friedmann

**Affiliations:** 1 Independent Scientist, PO Box 61571, Potomac, Maryland 20859 USA; 2 Division of Allergy & Immunology, University of South Florida College of Medicine, 13000 Bruce B. Downs Blvd, Tampa, Florida 33613 USA; 3 Organizational Systems and Adult Health, University of Maryland School of Nursing, 655 W. Lombard St., Baltimore, Maryland 21201 USA; 4 Present Address: The Hilltop Institute, University of Maryland Baltimore County, 1000 Hilltop Circle, Baltimore, Maryland 21250 USA

Correction to: *Scientific Reports* 10.1038/s41598-019-55259-8, published online 10 December 2019

This Article contains errors within the legends of Figure 2 and Figure 3.

The legend of Figure 2 is incorrect,

“Estimated impaired fecundity (number of pregnancy losses per 1000 woman-years) according to smoking status and number of pregnancies per thousand woman years, controlling for age, race/ethnicity, marital status, region, education, income, pelvic inflammatory disease, diabetes, hypertension, BMI, number of male partners and number of pregnancies as well as the interaction between asthma/hay fever status and number of pregnancies from the NSFG (N  =  9,284). Estimates based on complex samples analysis in Table 5 adjusted for multiple comparisons.”

should read:

“Estimated impaired fecundity (number of pregnancy losses per 1000 woman-years) according to smoking status and number of pregnancies per thousand woman years, controlling for age, race/ethnicity, marital status, region, education, income, pelvic inflammatory disease, diabetes, hypertension, BMI, number of male partners and number of pregnancies as well as the interaction between asthma/hay fever status and number of pregnancies from the NSFG (N  =  7,239). Estimates based on complex samples analysis in Table 5 adjusted for multiple comparisons.”

In addition, the legend of Figure 3,

“Estimated impaired fecundity (number of pregnancy losses per 1000 woman-years) according to asthma/hay fever status and number of pregnancies per thousand woman years, controlling for age, race/ethnicity, marital status, region, education, income, pelvic inflammatory disease, diabetes, hypertension, BMI, number of male partners, and number of pregnancies as well as the interaction of number of pregnancies with smoking from the NSFG (N  =  9,284). Estimates based on complex samples analysis in Table 5 adjusted for multiple comparisons.”

should read:

“Estimated impaired fecundity (number of pregnancy losses per 1000 woman-years) according to asthma/hay fever status and number of pregnancies per thousand woman years, controlling for age, race/ethnicity, marital status, region, education, income, pelvic inflammatory disease, diabetes, hypertension, BMI, number of male partners, and number of pregnancies as well as the interaction of number of pregnancies with smoking from the NSFG (N  =  7,239). Estimates based on complex samples analysis in Table 5 adjusted for multiple comparisons.”

